# LncRNA H19 over-expression inhibited Th17 cell differentiation to relieve endometriosis through miR-342-3p/IER3 pathway

**DOI:** 10.1186/s13578-019-0346-3

**Published:** 2019-10-15

**Authors:** Zheying Liu, Liya Liu, Yun Zhong, Mingbo Cai, Junbi Gao, Chaoyue Tan, Xiaoxiao Han, Ruixia Guo, Liping Han

**Affiliations:** Department of Gynecology, The First Affiliated Hospital of Zhengzhou University, No. 1 Jianshe Dong Road, Zhengzhou, 450052 People’s Republic of China

**Keywords:** LncRNA H19, miR-342-3p, IER3, Th17 cell differentiation, Endometriosis

## Abstract

**Objective:**

To investigate the mechanism of LncRNA H19 in Th17 cell differentiation and endometrial stromal cells (ESCs) proliferation in endometriosis (EMS).

**Methods:**

LncRNA H19, miR-342-3p and IER3 expressions were detected by qRT-PCR and western blot. The percentage of Th17 cells/CD4+ T cells was detected by flow cytometry. IL-17 level was measured by ELISA. The interaction of miR-342-3p and IER3 was confirmed by Luciferase reporter assay.

**Results:**

LncRNA H19 and IER3 expressions were down-regulated in mononuclear cells from peritoneal fluid (PFMCs) of patients with EMS or under Th17 differentiation conditions, whereas miR-342-3p expression was up-regulated and the percentage of Th17 cells was increased in PFMCs of patients with EMS or under Th17 differentiation conditions. Over-expression of LncRNA H19 decreased IL-17 level and the percentage of Th17 cells/CD4+ T cells. Besides, we confirmed that miR-342-3p could target to IER3 and negatively regulate IER3 expression. LncRNA H19 over-expression suppressed Th17 differentiation and ESC proliferation through regulating miR-342-3p/IER3. In vivo experiments showed LncRNA H19 over-expression suppressed the growth of Th17 cell differentiation-induced endometriosis-like lesions.

**Conclusion:**

LncRNA H19 was down-regulated in PFMC of patients with EMS or under Th17 polarizing conditions, and LncRNA H19 over-expression suppressed Th17 cell differentiation and ESCs proliferation through miR-342-3p/IER3 pathway.

## Introduction

Endometriosis (EMS) is a common gynecological disorder characterized by the presence of endometrial tissue outside the uterine cavity, which affects approximately 10% of reproductive-aged women and causes pain and infertility [1]. Although this disease has been investigated for decades, the pathogenesis of EMS is still not fully understood. Sikora et al. have found that retrograde menstruation forms endometriosis on the basis of endometrial fragments reaching the pelvis via transtubal retrograde flow and causes chronic inflammation [2]. T helper cell 17 (Th17) is a subset of T cells differentiated from CD4+ T cells that can secrete interleukin 17 (IL-17) and play important roles in autoimmune disease and defensive response [3]. IL-17 family members can induce inflammation, activate dendritic cells or macrophages, and promote tissue inflammation [4]. Researchers studied the phenotype of Th17 cells and found an elevation of inflammatory cytokines IFN-γ, IL-17A, TNF-α, IL-1β in the peritoneal fluid (PF) of patients with endometriosis, and the percentage of Th17 cells was remarkably increased in S (I-II) and S (III-IV) stages of EMS [5]. Therefore, we focus on Th17 cells and try to find the signaling pathways that regulate Th17 cells in EMS.

Immediate early response gene (IER3), also called IEX-1, belongs to the group of genes rapidly activated during inflammation that functions as an apoptosis inhibitor in TNF-induced apoptosis and extends duration of immune responses [6, 7]. Zhi et al. proved that lack of IEX-1 promoted Th17 differentiation and increased IL-17 production [8]. So, we assume that IER3 might involve in the signaling pathways that regulate Th17 cells in EMS. MicroRNAs act as a post-transcriptional regulatory function through binding to the 3′-untranslated region (UTR) of the target genes in EMS [9, 10]. It has been found that miR-342-3p is highly expressed in serum of woman with EMS through microarray profiling [11]. Bioinformatics software predicts IER3 is one of the target genes of miR-342-3p. Therefore, miR-342-3p may involve in the regulation of Th17 cells in EMS through targeting IER3.

Long non-coding RNAs are RNAs longer than 200 nucleotides that involve in post-transcriptional and epigenetic regulation in various biological processes [12, 13]. LncRNA H19 is one of the most widely studied LncRNAs in cancers, angiogenesis, diabetes mellitus, etc. [14, 15]. In 2010, H19 was firstly detected in women with EMS and found lower expression in endometrial tissue of infertility cases than fertile cases [16]. Recently, researchers have found H19 expression is remarkably decreased in the eutopic endometrium of women with EMS, and discover its mechanism in reducing the proliferation of endometrial stromal cells (ESCs) [17]. Moreover, Wang et al. proved that H19 interacted with miR-342-3p and negatively modulated miR-342-3p in gallbladder cancer. Hence, we speculate LncRNA H19/miR-342-3p/IER3 is involved in the regulation of Th17 cells in EMS.

In this study, we showed that LncRNA H19 was down-regulated in mononuclear cells from peritoneal fluid (PFMCs) of patients with EMS, and found over-expression of H19 decreased the secretion of IL-17 and reduced the percentage of Th17 cells/CD4+ T cells through miR-342-3p/IER3, thereby contributing to suppress Th17 differentiation and ESC proliferation.

## Materials and methods

### Preparation of peritoneal fluid samples

Peritoneal fluid samples were collected from patients with regular menstrual cycles who underwent a salpingo-oophorectomy or evisceration for the treatment of ovarian endometriotic cysts without any hormonotherapy for at least 3 months prior to operation (EMS group, n = 20) and premenopausal patients who underwent hysterectomies for subserousal leiomyoma without evidence of endometriosis (control group, n = 16) under sterile condition after the laparoscopy in order to minimize blood contamination. The protocols were approved by the Ethics Committee of The First Affiliated Hospital of Zhengzhou University (2019-KY-197). All patients signed informed consent.

### Preparation and identification of PFMCs

PFMCs were prepared and identified according to previous report [18]. Peritoneal fluid samples collected from EMS and control groups as described above were centrifuged at 200*g* for 5 min, and the supernatant was removed. Cell pellets were re-suspended in phosphate buffered saline (PBS), and isolated by Histopaque-1077 (Sigma, USA) according to the manufacturer’s instructions. Cells were centrifuged at 150*g* for 30 min, and collected at the interface. For the identification of PFMCs (purity > 97%), indirect immunofluorescence (IIF) was conducted. Anti-CD3 (Abcam, USA), anti-B19 (Abcam, USA), anti-CD56 (Invitrogen, USA), and anti-CD14 (Abcam, USA) monoclonal antibodies were used to identify T lymphocytes, B lymphocytes, natural killer lymphocytes, and macrophages.

### Isolation and purification of CD4+ T cells

Peripheral blood mononuclear cells (PBMCs) were collected from healthy fertile women and isolated by Histopaque-1077 (Sigma, USA) according to the manufacturer’s instructions, washed twice with RPMI-1640 medium (Gibco, USA), counted by a Neubauer hemocytometer, and re-suspended at 1 × 10^6^ cells/mL. MagniSort™ Human CD4 T cell Enrichment Kit (Invitrogen, USA) was used to isolate CD4+ T cells according to the manufacturer’s instructions (purity > 95%). Naïve CD4+ T cells were isolated using MagniSort Human CD4 Naive T cell Enrichment Kit (eBioscience, USA). The protocols were approved by the Ethics Committee of The First Affiliated Hospital of Zhengzhou University. All patients signed informed consent.

For CD4+ T cell transfection, lentivirus-mediated H19 over-expression (lenti-H19), lentivirus-mediated miR-342-3p mimic, lentivirus-mediated miR-342-3p inhibitor lentiviral vectors and scramble sequence was set as negative control.

### Th17 polarization induction

CD4+ T cells differentiation into Th17 cells were performed according to previous report [19]. CD4+ T cells (5 × 10^5^) were incubated for 48 h with anti-CD3 (1 μg/mL) (Abcam, USA), anti-CD28 antibody (1 μg/mL) (Abcam, USA), IL-1β (20 ng/mL)(Gibco, USA), IL-6 (20 ng/mL) (Gibco, USA), IL-23 (20 ng/mL) (Invitrogen, USA), IFN-γ-neutralizing antibody (2 μg/mL) (Cell Signaling Technology, USA), and IL-4-neutralizing antibody (2 μg/mL) (Cell Signaling Technology, USA).

### Quantitative real-time RCR (qRT-PCR)

Total RNAs from PFMCs and CD4+ T cells were extracted by Trizol (Invitrogen, USA), and inversely transcribed into cDNA using the High-Capacity cDNA archive kit (Invitrogen, USA). qRT-PCR was conducted to measure H19 and miR-342-3p expression using PowerUp™ SYBR™ Green Master Mix (Invitrogen, USA). The relative expressions of H19 and miR-342-3p were expressed as a function of threshold cycle (Ct) and analyzed by 2^−ΔΔCt^ method. Specific primers for H19 and miR-342-3p were as follows: H19, F: 5′-GCTCCACTGACCTTCTAAAC-3′; miR-342-3p, F: 5′-UCUCACACAGAAAUCGCACCCGU-3′.

### Western blot

PFMCs and CD4+ T cells were lysed in Radio Immunoprecipitation Assay (RIPA) buffer (Beyotime, China). Protein samples (50 ng) was separated by SDS-polyacrylamide gel electrophoresis (PAGE) and transferred to polyvinylidene fluoride (PVDF) membrane (Invitrogen, USA). The membrane was incubated with primary antibodies against IER3 (Invitrogen, USA), β-actin (Abcam, USA) and horseradish peroxidase-conjugated secondary antibody (Abcam, USA). Blots were detected by enhanced chemiluminescence, and band intensities were quantified using image software Image Lab (Bio-Rad, USA). β-actin was used as an internal control.

### Flow cytometry

PFMCs or CD4+ T cells were collected and re-suspended at 2 × 10^6^ cells/mL. Cells were detected by BD FACSCanto II flow cytometry (BD, USA) and analyzed using CELLQuest software. Cells positive for both CD4 and intercellular IL-17A were considered as Th17. Cells were collected and incubated with APC-conjugated anti-CD4 antibody (Invitrogen, USA), anti-IL-17A antibody (Invitrogen, USA) and anti-IFN-γ (Invitrogen, USA) for the observation of Th17 cells.

### Enzyme-linked immuno sorbent assay (ELISA)

The cytokine IL-17 level from CD4+ T cell culture supernatant was detected by the IL-17A Human ELISA Kit (Invitrogen, USA).

### Luciferase reporter assay

The sequence of IER3 3′UTR or the mutated sequence was predicted to interact with miR-342-3p and inserted into pGL3 vector (Promega, USA) which were noted as IER3 3′UTR wild-type (WT) or IER3 3′UTR mutant (MUT) that constructed by Sangon Biotech (Shanghai, China). The reporter plasmid (IER3 3′UTR WT or IER3 3′UTR MUT, 500 ng) and microRNAs (miR-342-3p mimic, miR-342-3p inhibitor, or negative control, 1000 ng) were transfected into HEK293 cells for 48 h for Luciferase reporter assay. Luciferase activity was measured by dual Glo™ Luciferase Assay System (Promega).

### Isolation and culture of ESC

ESCs were isolated and cultured according to previous report [5]. Endometrial tissues were collected from patients with regular menstrual cycles but without endometriosis and/or adenomyosis who underwent a hysterectomy for the treatment of uterine leiomyoma under sterile conditions and none of the included patients had experienced hormonotherapy. Tissues were digested with 0.1% collagenase type IV (Sigma, USA) at 37 °C for 30 min with constant agitation. Then, sterile gauzes (pore diameter size: 200 mesh) was used to filtrate the tissue pieces to remove debris. The supernatant was discarded during gentle centrifugation, and the cells were re-suspended in DMEM/F-12 medium (Gibco, USA). For removal of epithelial cells, ESCs were passed through sterile gauzes (pore diameter size: 400 mesh). For removal of leukocytes and erythrocytes, the filtrated suspension was layered over Ficoll, and centrifuged at 800×*g* for 20 min. The middle layer was collected and washed with D-Hanks solution. Then, ESCs were placed in a culture flask, and allowed to adhere for 20 min. The adherent stromal cells were cultured as monolayer in flasks with DMEM/F-12 (Gibco, USA) containing 10% Fetal bovine serum (Gibco, USA), and incubated in 5% CO_2_ incubator at 37 °C. The protocols were approved by the Ethics Committee of The First Affiliated Hospital of Zhengzhou University. All patients signed informed consent.

### Co-culture of CD4+ T cells and ESC

To establish ESC and CD4+ T cells co-culture unit, ESCs were cultured in 48-well plates at a concentration of 2 × 10^5^ cells/well until they adhered to the plastic. Then, the media were removed, and CD4+ T cells with different treatment were applied over ESCs at the same concentration for 48 h.

### MTT assay

3-(4,5-dimethylthiazol-2-yl)-2,5-diphenyltetrazolium bromide (MTT) assay was used to detect ESC proliferation. ESCs (2 × 10^4^) were seeded into a 96-well plate and incubated overnight. 20 μL MTT (5 mg/mL; Invitrogen, USA) was added to each well and cultured for 4 h. Then, cells were lysed using dimethylsulfoxide (150 μL/well; Sinopharm Chemical Reagent, China). The optical density was read at 570 nm.

### Resazurin assay

Resazurin assay was also used to detect ESC proliferation according to previous report [20]. ESCs (2 × 10^4^) were seeded into a 96-well plate and incubated overnight. 20 μL resazurin solution (0.1 mg/mL; Invitrogen, USA) was added to each well and cultured at 37 °C for 4 h. Fluorescence intensity was monitored, the excitation at 530 nm and emission at 590 nm was measured using a microplate reader (Thermo Scientific, USA).

### Intraperitoneal endometriosis model

The nude mouse endometriosis model was established according to previous report [5]. The nude mice (8 week-old) and female C57BL/6 mice were purchased from Laboratory animal center of Zhengzhou University. The animal experiments were approved by Ethics Committee of The First Affiliated Hospital of Zhengzhou University. Nude mice were used to construct an allotransplantation of intraperitoneal endometriosis model. At 0th day, the uterus of female C57BL/6 mice was minced, and the tissue debris was intraperitoneally injected into the nude mice. At 5th day, CD4+ T cells from female C57BL/6 mice were transfected with lenti-NC or lenti-H19, induced for Th17 polarization and transferred to the abdominal cavity in endometriosis nude mice. So the nude mice were divided into EMS, EMS + Th17, EMS + lenti-NC + Th17, and EMS + lenti-H19 + Th17 groups, with six mice in each group. At 14th day, the nude mice were sacrificed, and the endometriosis-like lesions and PF were collected for the detection of lesion weight, and LncRNA H19, miR-342-3p and IER3 expressions.

### Statistical analysis

All data were presented as mean ± standard deviation and analyzed by SPSS software (Version 15.0, USA). Each experiment was repeated for three times. The differences between groups were assessed by t-test or one-way analysis of variance (ANOVA), with p < 0.05 considered statistically significant.

## Results

### LncRNA H19 was down-regulated in PFMC of patients with EMS

**Fig. 1:**
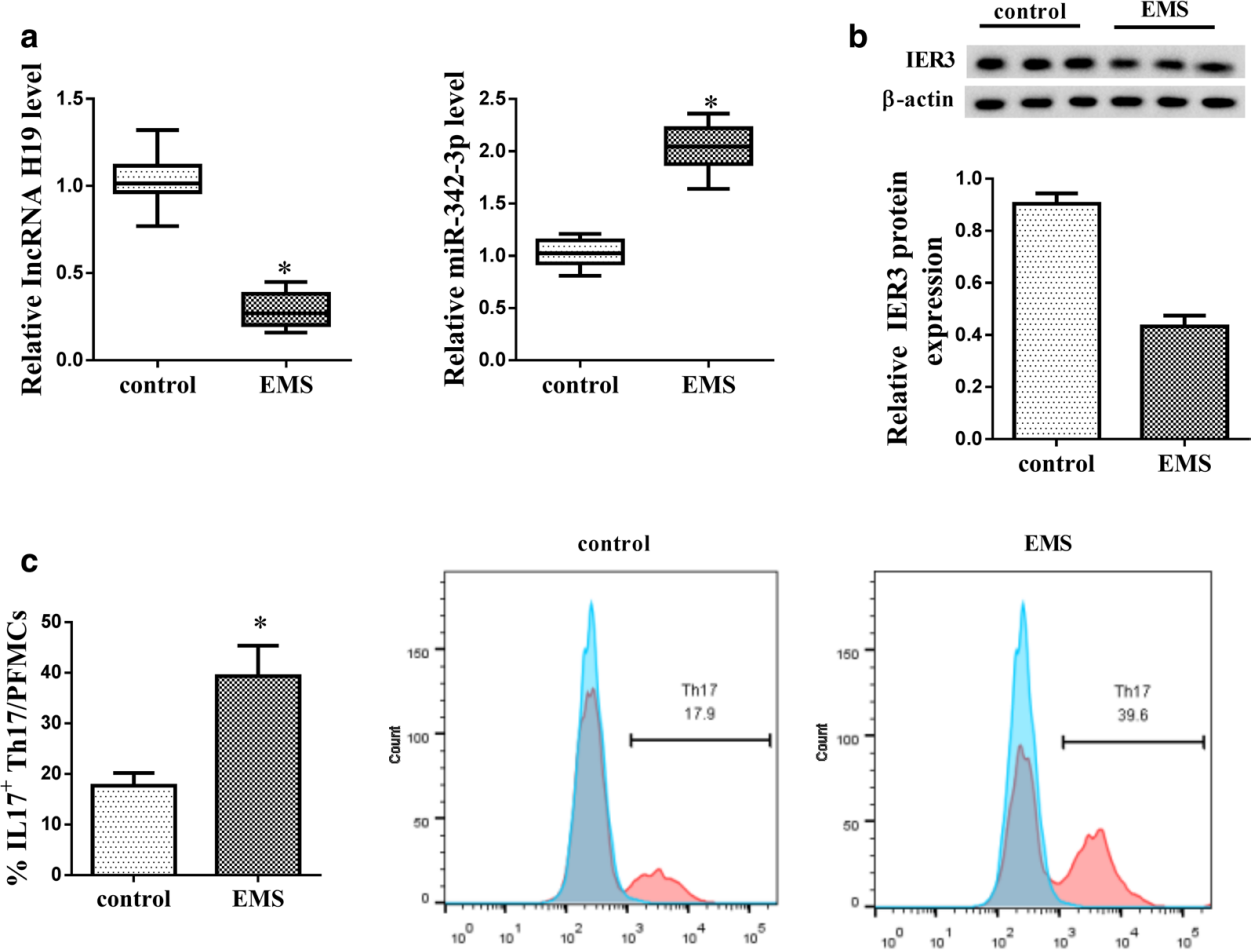
LncRNA H19 was down-regulated in mononuclear cells from peritoneal fluid (PFMC) of patients with endometriosis (EMS). PFMCs were isolated from patients with EMS (n = 20) and controls (n = 16). **a** qRT-PCR showed that LncRNA H19 was down-regulated and miR-342-3p was up-regulated in PFMC of EMS group than that of control group. **b** Western blot showed protein level of IER3 was down-regulated in PFMC of EMS group. **c** Flow cytometry showed the percentage of IL17 + Th17/PMSCs was higher in PFMC of EMS group than that of control group. *p < 0.05, compared with control

We first examined the expressions of LncRNA H19, miR-342-3p and IER3 in PFMCs in patients with EMS. As shown in Fig. 1a, LncRNA H19 expression was significantly down-regulated in PFMC of EMS group than that of control group, and miR-342-3p expression was significantly up-regulated in PFMC of EMS group than that of control group. Protein level of IER3 was significantly down-regulated in PFMC of EMS group than that of control group (Fig. 1b, Additional file 1: Figure S1B). We also observed the percentage of IL17 + Th17 cells in PFMCs was higher in EMS group than that of control group (Fig. 1c).

### LncRNA H19 was down-regulated in Th17 differentiation conditions

**Fig. 2:**
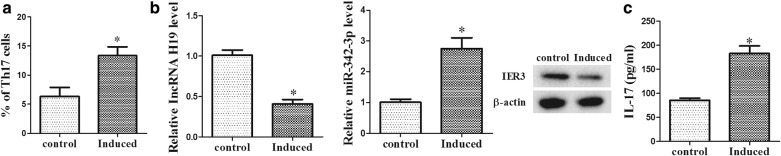
LncRNA H19 was down-regulated in Th17 polarizing conditions. CD4+ T cells from healthy patients were incubated under appropriate conditions to induce Th17 polarizing for 48 h. **a** The percentage of Th17 cells/CD4+ T cells was higher in induced group than that of control group. **b** LncRNA H19 was down-regulated, miR-342-3p was up-regulated and IER3 was down-regulated in induced group than that of control group. **c** ELISA assay showed that IL-17 level was increased in induced group than that of control group. *p < 0.05, compared with control

Next, we isolated CD4+ T cells from healthy patients and induced Th17 polarization for 48 h under Th17 polarized conditions. After confirming the induction is successful, we tested the expressions of H19, miR-342-3p and IER3. We found the percentage of Th17 cells/CD4+ T cells was significantly increased in induced group than that of control group (Fig. 2a). The percentage of Th17 cells/naïve CD4+ T cells was also significantly increased in induced group than that of control group (Additional file 1: Figure S1A). LncRNA H19 expression was significantly down-regulated, miR-342-3p expression was significantly up-regulated, and IER3 protein level was significantly down-regulated in induced group than that of control group (Fig. 2b). IL-17 level was also significantly increased in induced group than that of control group (Fig. 2c).

### LncRNA H19 over-expression suppressed Th17 differentiation

**Fig. 3:**
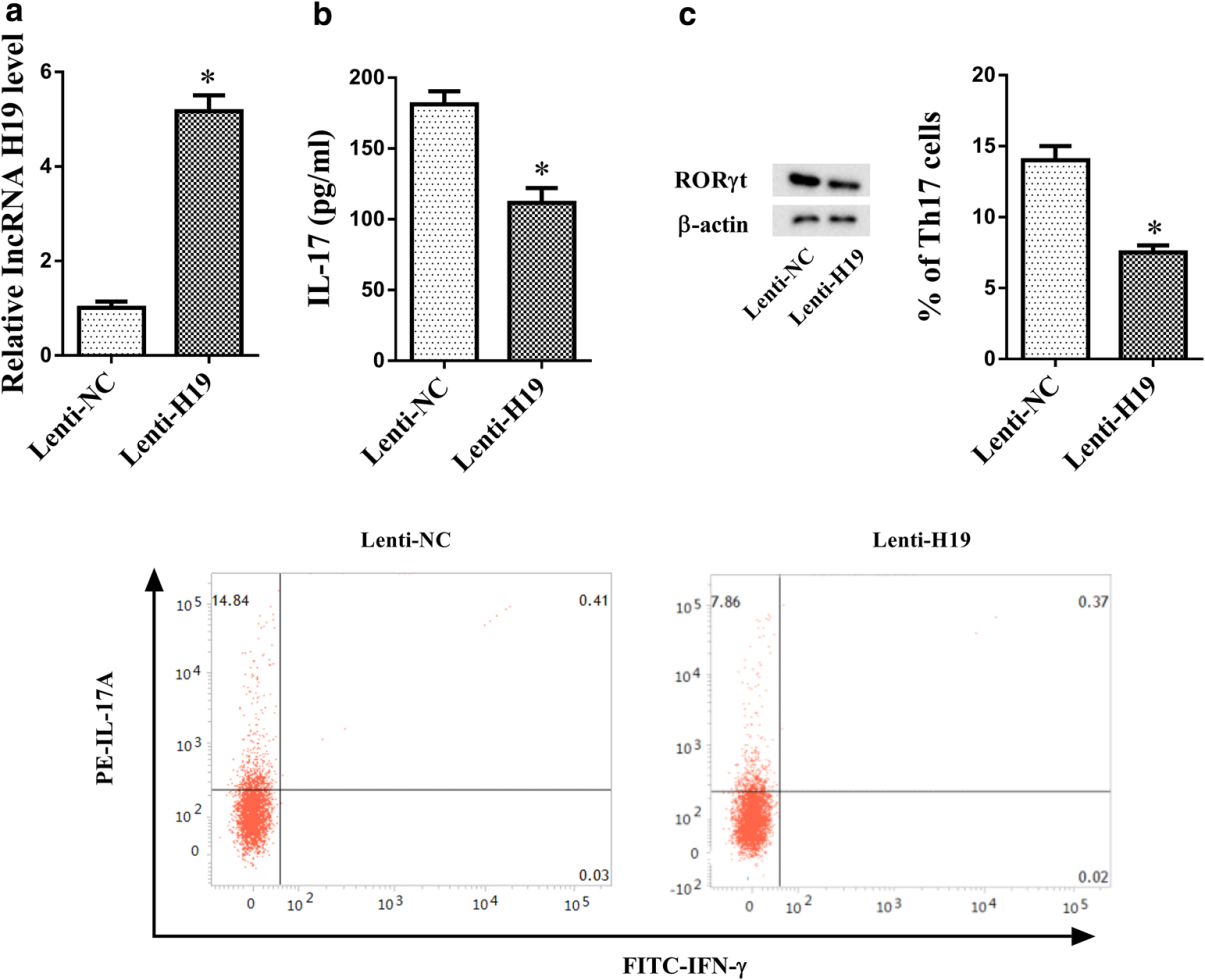
LncRNA H19 over-expression suppressed Th17 differentiation. CD4+ T cells were transfected with H19 over-expression lentiviral vectors or negative controls. **a** LncRNA H19 was up-regulated in CD4+ T cells transfected with H19 over-expression lentiviral vectors. **b** IL-17 level was decreased in CD4+ T cells transfected with H19 over-expression lentiviral vectors. **c** TH17 cell marker RORγt expression in Lenti-NC and Lenti-H19 groups was detected by western blot. The percentage of Th17 cells/CD4+ T cells was decreased in lenti-H19 group. *p < 0.05, compared with lenti-NC

To observe the effect of LncRNA H19 on Th17 differentiation, CD4+ T cells were transfected with H19 over-expression lentiviral vectors. As shown in Fig. 3a, LncRNA H19 expression was significantly up-regulated in CD4+ T cells transfected with H19 over-expression lentiviral vectors than that of Lenti-NC group. IL-17 level was significantly decreased in lenti-H19 group than that of Lenti-NC group (Fig. 3b). Then, CD4+ T cells in Lenti-NC group and Lenti-H19 group were under an induced Th17 polarizing condition. We found TH17 cell marker RORγt expression was down-regulated in Lenti-H19 group than Lenti-NC group (Fig. 3c), and the percentage of Th17 cells/CD4+ T cells was decreased in lenti-H19 group than Lenti-NC group (14.84% vs 7.86%) (Fig. 3c). These data proved that LncRNA H19 over-expression suppressed Th17 differentiation.

### LncRNA H19 over-expression regulated miR-342-3p/IER3 expression

**Fig. 4:**
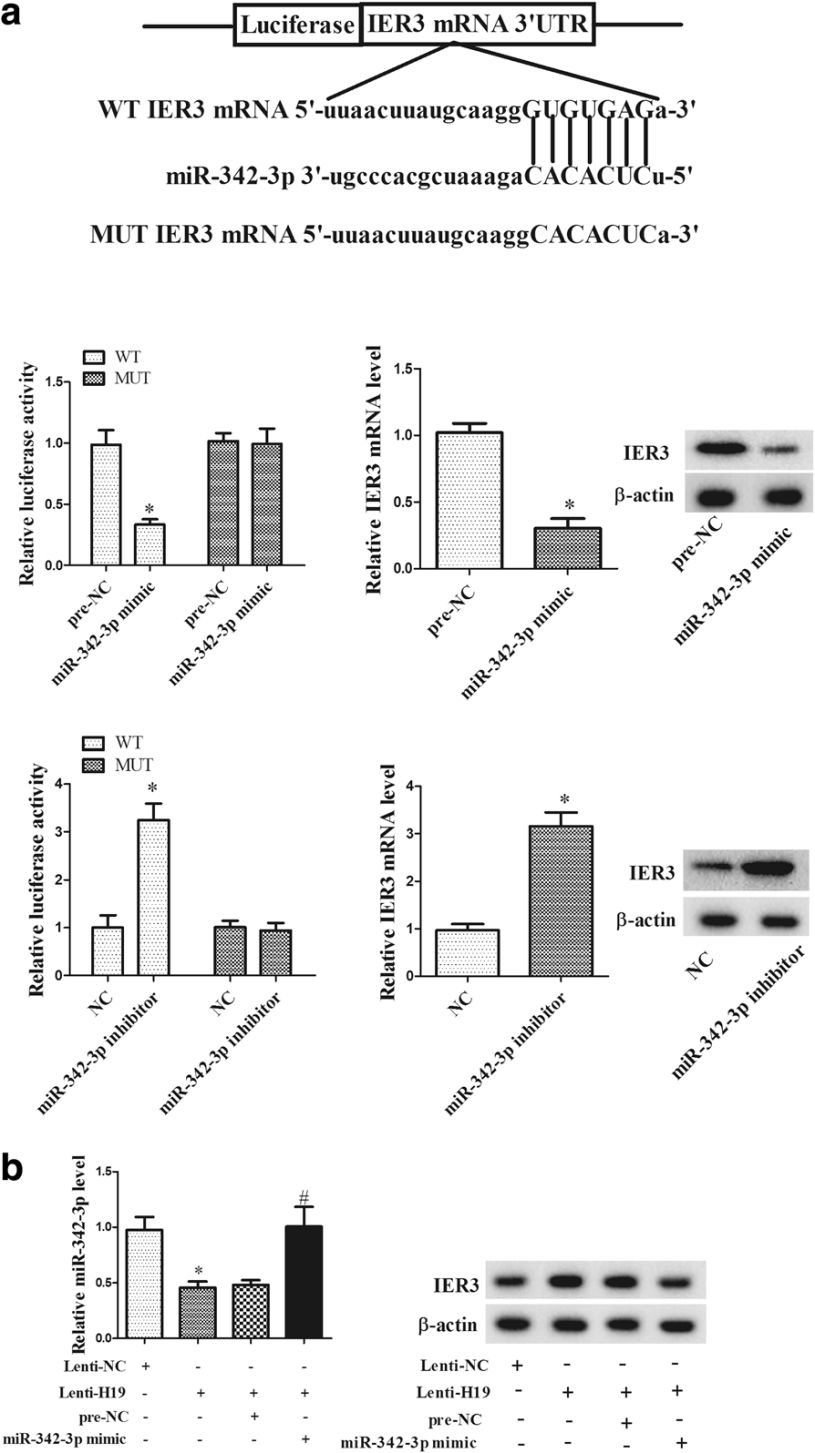
LncRNA H19 over-expression regulated miR-342-3p/IER3 expression. **a** Bioinformatics software predicted the miR-342-3p targeted to 3′UTR of IER3. miR-342-3p mimic or inhibitor were transfected into HEK293 cells to detect the luciferase activity. IER3 mRNA and protein levels were detected by qRT-PCR or Western blot after miR-342-3p mimic or inhibitor treatment. **b** CD4+ T cells were divided into lenti-NC, lenti-H19, lenti-H19 + pre-NC, lenti-H19 + miR-342-3p mimic groups. miR-342-3p expression and IER3 protein level were detected by qRT-PCR and Western blot. *p < 0.05, compared with pre-NC or lenti-NC; ^#^p < 0.05, compared with lenti-H19 + pre-NC

According to the prediction of bioinformatics software, miR-342-3p could target to 3′UTR of IER3 (Fig. 4a). Next, miR-342-3p mimic or inhibitor was transfected into HEK293 cells to detect the luciferase activity. miR-342-3p mimic significantly decreased the luciferase activity of IER3-WT, and there was no significant change in the luciferase activity of IER3-MUT. miR-342-3p inhibitor significantly increased the luciferase activity of IER3-WT, and there was no significant change in the luciferase activity of IER3-MUT. mRNA and protein level of IER3 was down-regulated by miR-342-3p mimic, and up-regulated by miR-342-3p inhibitor. These findings indicated that miR-342-3p targeted to 3′UTR of IER3 and negatively regulated IER3 expression. We further investigated the role of LncRNA H19 over-expression in miR-342-3p and IER3 expression in CD4+ T cells. We found that Lenti-H19 significantly decreased miR-342-3p expression, and miR-342-3p mimic reversed the inhibition effect of Lenti-H19. Lenti-H19 increased IER3 protein level, and miR-342-3p mimic reversed the promotion effect of Lenti-H19 (Fig. 4b).

### LncRNA H19 over-expression suppressed Th17 differentiation and ESC proliferation through miR-342-3p/IER3

**Fig. 5:**
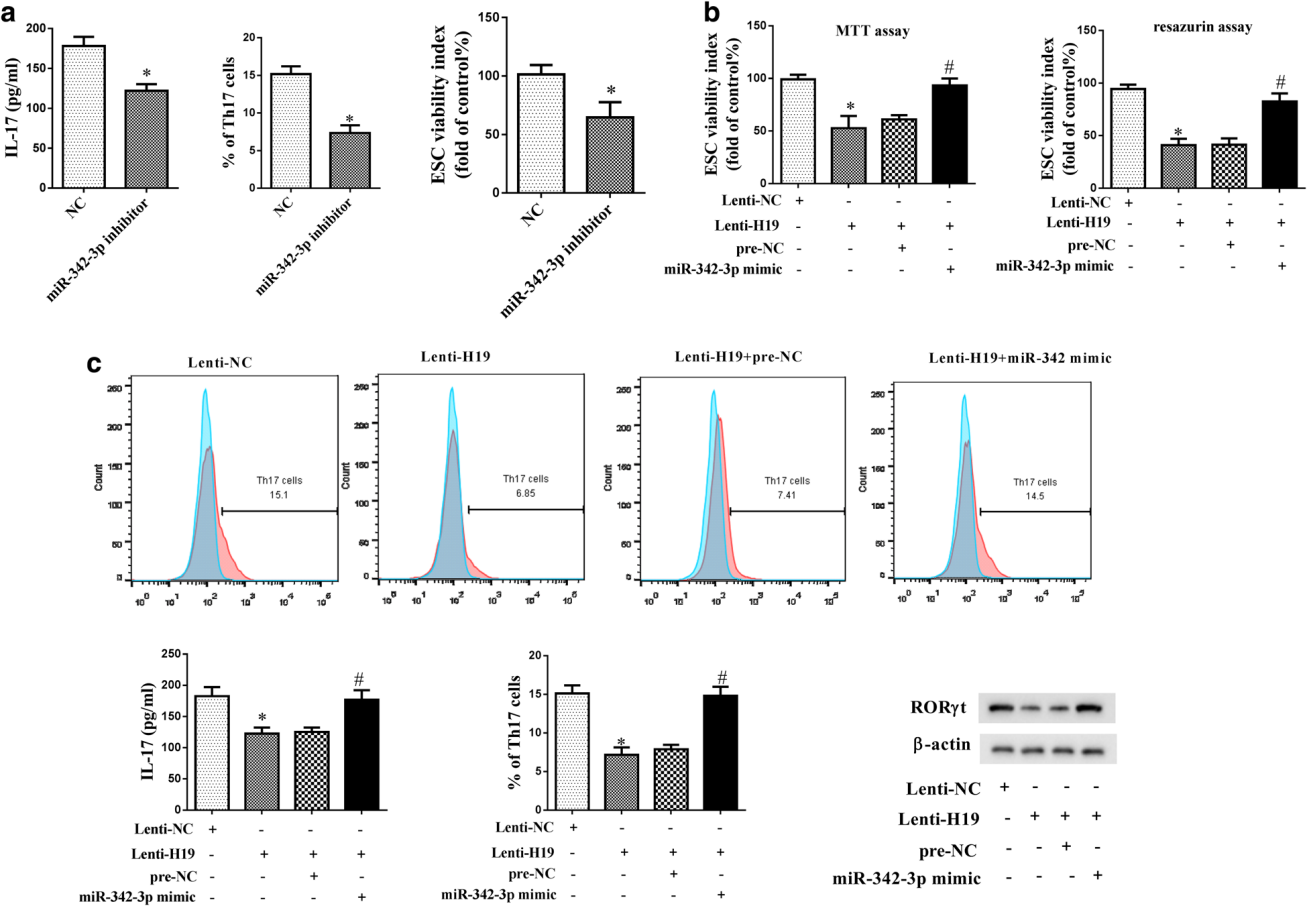
LncRNA H19 over-expression suppressed Th17 differentiation and ESC proliferation through miR-342-3p/IER3. **a** miR-342-3p inhibitor suppressed IL-17 level, the percentage of Th17 cells/CD4+ T cells, and ESC viability. **b** CD4+ T cells were divided into lenti-NC, lenti-H19, lenti-H19 + pre-NC, lenti-H19 + miR-342-3p mimic groups. Then, CD4+ T cells were co-cultured with ESC for 48 h. The viability of ESC was detected by MTT assay and resazurin assay. **c** IL-17 level was detected using ELISA. The percentage of Th17 cells/CD4+ T cells was detected using flow cytometry. TH17 cell marker RORγt expression was detected by western blot. *p < 0.05, compared with NC or lenti-NC; ^#^p < 0.05, compared with lenti-H19 + pre-NC

We went on to investigate the signaling pathway of LncRNA H19 in the regulation of Th17 differentiation and ESC proliferation. CD4+ T cells in NC and miR-342-3p groups were under an induced Th17 polarizing condition. miR-342-3p inhibitor significantly decreased IL-17 level, reduced the percentage of Th17 cells/CD4+ T cells, and suppressed ESC viability (Fig. 5a). CD4+ T cells in Lenti-NC, Lenti-H19, Lenti-H19 + pre-NC, Lenti-H19 + miR-342-3p mimic groups were also under an induced Th17 polarizing condition. Then, CD4+ T cells were co-cultured with ESCs for 48 h. MTT assay and resazurin assay showed that Lenti-H19 significantly suppressed ESC viability, and miR-342-3p mimic reversed the inhibition effect of H19 over-expression (Fig. 5b). In addition, Lenti-H19 significantly decreased IL-17 level, reduced the percentage of Th17 cells/CD4+ T cells, and down-regulated TH17 cell marker RORγt expression, and miR-342-3p mimic reversed the inhibition effect of H19 over-expression (Fig. 5c).

### LncRNA H19 over-expression inhibited the ectopic growth of endometriosis-like lesions in the nude mouse endometriosis model

**Fig. 6:**
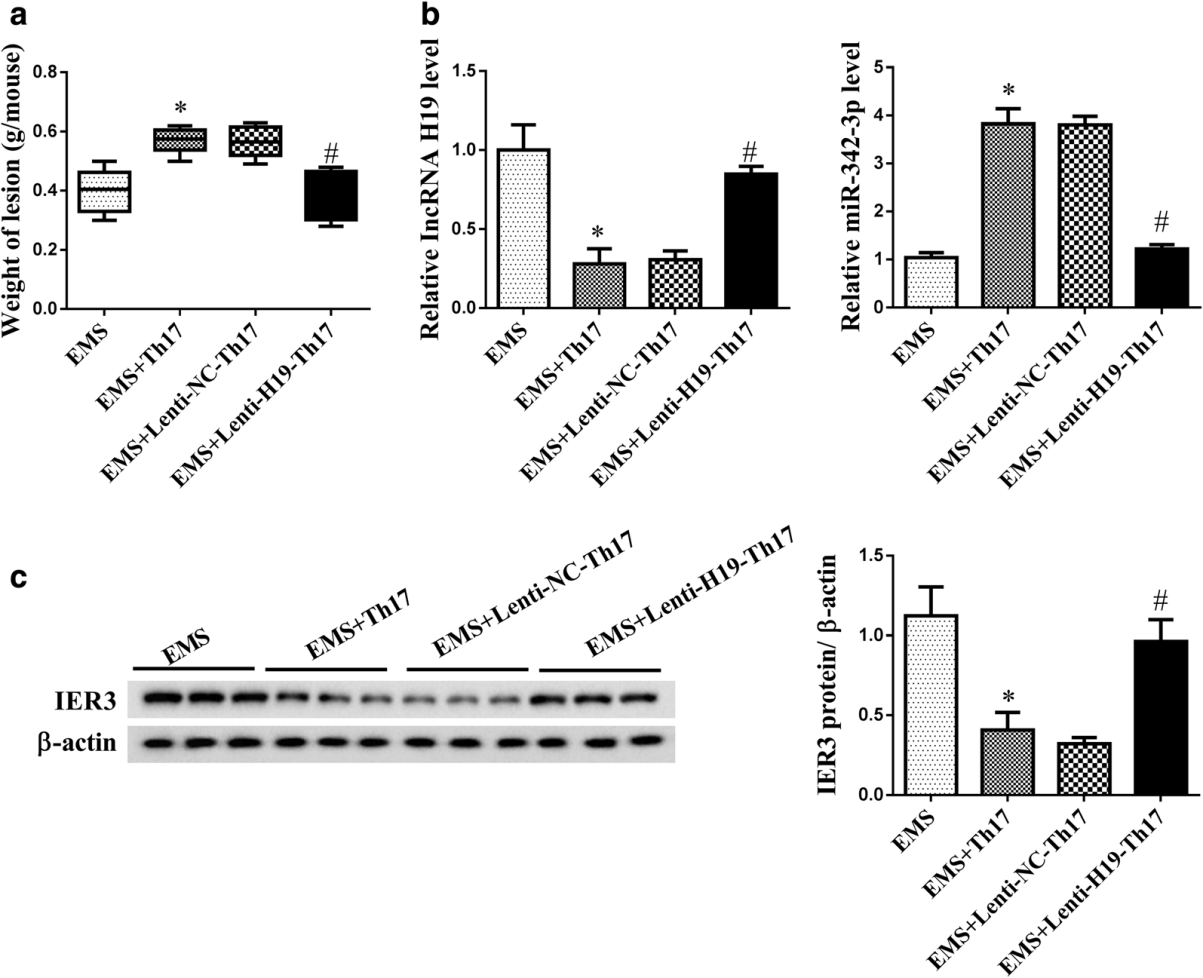
LncRNA H19 over-expression inhibited the ectopic growth in the nude mouse endometriosis model. The nude mouse endometriosis model was established. CD4+ T cells were transfected with LncRNA H19 over-expression lentiviral vectors and induced Th17 polarizing, then intraperitoneally injected into the nude mouse endometriosis model. Nude mice were divided into EMS, EMS + Th17, EMS + lenti-Th17, EMS + lenti-H19-Th17 groups, with six mice in each group. **a** The weight of endometriosis-like lesions from nude mouse endometriosis model was increased in EMS + Th17 group, and H19 over-expression reversed the promotion effect of EMS + Th17. **b** Peritoneal fluid (PF) was collected to detect H19 and miR-342-3p expressions. LncRNA H19 was down-regulated in PF of EMS + Th17 group, and H19 over-expression reversed the inhibition effect of EMS + Th17. miR-342-3p was up-regulated in PF of EMS + Th17 group, and H19 over-expression reversed the promotion effect of EMS + Th17. **c** IER3 was down-regulated in PF of EMS + Th17 group, and H19 over-expression reversed the inhibition effect of EMS + Th17. *p < 0.05, compared with EMS; ^#^p < 0.05, compared with EMS + lenti-NC + Th17

Studies have reported that Th17 differentiation promoted the proliferation and invasion of ESC, thus to accelerate the growth and implantation of the endometriosis-like lesions [5]. At the 14th day after the establishment of the nude mouse endometriosis model, the nude mice were sacrificed and the ectopic lesions were obtained. We observed the weight of endometriosis-like lesions from nude mouse endometriosis model was increased in EMS + Th17 group, and H19 over-expression decreased the weight of endometriosis-like lesions compare to EMS + Lenti-NC-Th17 group (Fig. 6a). LncRNA H19 was down-regulated in PF of EMS + Th17 group, and H19 over-expression increased LncRNA H19 expression compare to EMS + Lenti-NC-Th17 group (Fig. 6b). miR-342-3p expression was up-regulated in PF of EMS + Th17 group, and H19 over-expression down-regulated miR-342-3p expression compare to EMS + Lenti-NC-Th17 group (Fig. 6b). Protein level of IER3 was down-regulated in PF of EMS + Th17 group, and H19 over-expression up-regulated IER3 level compare to EMS + Lenti-NC-Th17 group (Fig. 6c).

## Discussion

This study investigates the role of LncRNA H19 in Th17 cell differentiation and ESC proliferation, and its interaction with miR-342-3p/IER3 signaling pathway in EMS. The data showed that patients with EMS had decreased LncRNA H19 and IER3 expressions, and increased miR-342-3p expression and Th17 cells/CD4+ T cells percentage compared with those without EMS. Under Th17 polarizing condition, we found LncRNA H19 and IER3 expressions were down-regulated, and miR-342-3p expression and IL-17 secretion were up-regulated compared with control group. We showed that LncRNA H19 over-expression could decrease IL-17 secretion, suppress Th17 differentiation and inhibit ESC proliferation through inhibiting miR-342-3p. In vivo experiments strongly indicated a suppression effect of H19 over-expression on Th17 differentiation and it inhibition on the growth of endometriosis-like lesions.

EMS is characterized by the presence of ectopic endometrium that causes pain, infertility and lesion progression. The pain can be controlled by medical or surgical treatment [21]. However, lesions cannot be eradicated despite the pain can be managed by the pharmacological inhibition of ovulation and menstruation, and the benefit from surgery is often temporary [22]. The recurrence of symptoms and lesion is between 40 and 50% at a 5-year follow-up period [23]. Hence, it is meaningful to find effective treatment for EMS. The balance of Th17 and regulatory T cells is important in many immunological pathologies, and it has been reported that IL-17A produced by Th17 cells was highly expressed in the EMS lesion and could contribute to disease progression in triggering proinflammatory cytokines and angiogenic growth factors [24]. Recent study found that in severe EMS, the percentage of Th17 cells in PF was higher than that of early (I/II stage) EMS, which indicated the increase of Th17 percentage in peritoneal fluid was related with the severity of EMS [24]. In this study, we showed that the percentage of Th17 cells was increased in PFMC of patients with EMS, which was consistent with previous report [25].

Numerous studies have reported that miRNAs could regulate Th17 cell differentiation through binding to the 3′-untranslated region (UTR) of the target genes [26, 27]. In this study, we observed miR-342-3p and IER3 were abnormally expressed in PFMC of patients with EMS or in Th17 polarizing conditions, and miR-342-3p knockdown suppressed IL-17 expression, the percentage of Th17 cells and ESC proliferation. Moreover, we first confirmed miR-342-3p could target to IER3 and negatively regulate IER3 expression.

A number of studies have suggested that LncRNAs can be abnormally expressed in EMS, and play roles in regulating stromal cell growth or estrogen receptor expression, which suggested that LncRNAs can be new biomarkers or novel therapeutic targets of EMS [28, 29]. Ghazal et al. showed a reduced expression of LncRNA H19 in the endometrium of women with EMS, and showed LncRNA H19 knockdown or over-expression negatively or positively affect ESCs proliferation via regulating Let-7/Igf1r expression [17]. In the present study, we showed LncRNA H19 was down-regulated in PFMC of patients with EMS or in Th17 polarizing conditions, and proved LncRNA H19 over-expression inhibited IL-17 expression and the percentage of Th17 cells by regulating miR-342-3p/IER3. Besides, LncRNA H19 over-expression suppressed the growth of Th17 cell differentiation-induced endometriosis-like lesions.

In conclusion, this study demonstrated that LncRNA H19 was down-regulated in PFMC of patients with EMS or under Th17 polarizing conditions, and LncRNA H19 over-expression suppressed Th17 cell differentiation and ESCs proliferation through miR-342-3p/IER3 pathway.

## Supplementary information

**Additional file 1: Figure S1.** A. The percentage of Th17 cells/CD4+ T cells and Th17 cells/naïve CD4+ T cells in control group and induced group was detected by flow cytometry. B. IER3 protein level in PFMC from control group and EMS group was detected by western blot. *p < 0.05, compared with control.

## Data Availability

Not applicable
