# Supplementary figures and images for: LncRNA H19 over-expression inhibited Th17 cell differentiation to relieve endometriosis through miR-342-3p/IER3 pathway
Source: Cell Biosci. 2019 Oct 15;9:84. doi: 10.1186/s13578-019-0346-3 (PMC6792244; doi:10.1186/s13578-019-0346-3)

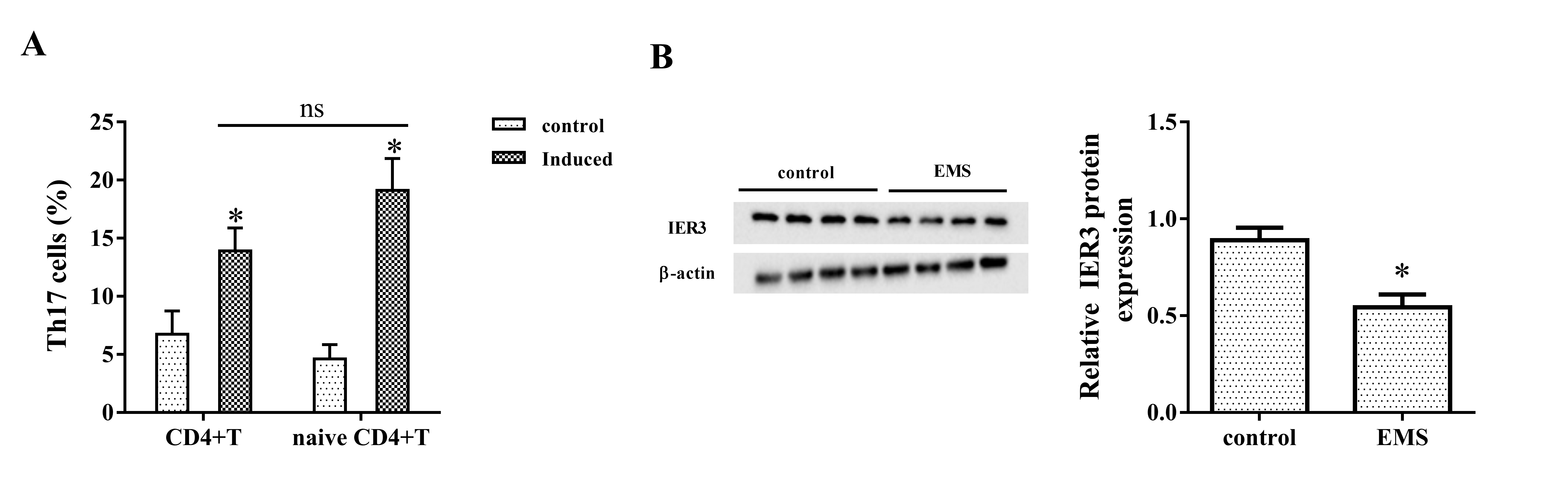

Supplement: Supplementary file 1 — Additional file 1: Figure S1. A. The percentage of Th17 cells/CD4+ T cells and Th17 cells/naïve CD4+ T cells in control group and induced group was detected by flow cytometry. B. IER3 protein level in PFMC from control group and EMS group was detected by western blot. *p < 0.05, compared with control. [file 13578_2019_346_MOESM1_ESM.tif]
